# Formation of Catalytic Hotspots in ATP-Templated Assemblies

**DOI:** 10.1021/jacs.2c09343

**Published:** 2022-12-28

**Authors:** Krishnendu Das, Haridas Kar, Rui Chen, Ilaria Fortunati, Camilla Ferrante, Paolo Scrimin, Luca Gabrielli, Leonard J. Prins

**Affiliations:** Department of Chemical Sciences, University of Padova, Via Marzolo 1, 35131 Padova, Italy

## Abstract

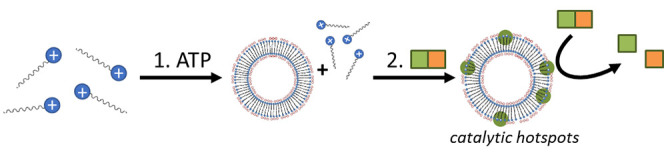

The self-assembly of surfactant-based structures that rely for their formation on the combination of a thermodynamically controlled and a dissipative pathway is described. Adenosine triphosphate (ATP) acts as a high-affinity template and triggers assembly formation at low surfactant concentrations. The presence of these assemblies creates the conditions for the activation of a dissipative self-assembly process by a weak-affinity substrate. The substrate-induced recruitment of additional surfactants leads to the spontaneous formation of catalytic hotspots in the ATP-stabilized assemblies that cleave the substrate. As a result of the two self-assembly processes, catalysis can be observed at a surfactant concentration at which low catalytic activity is observed in the absence of ATP.

## Introduction

**Figure 1:**
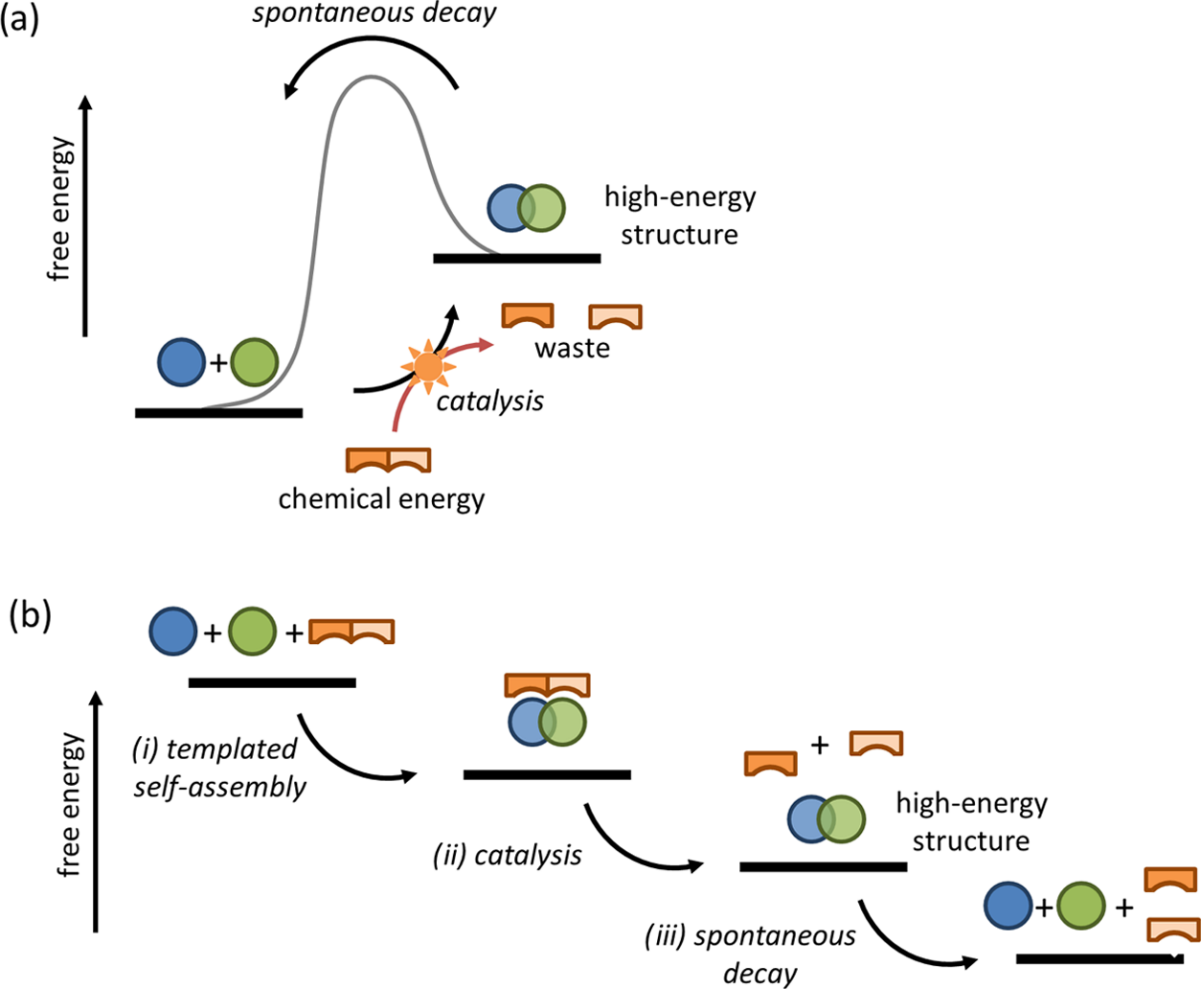
(a) Exploitation of chemical energy to form a high-energy structure. (b) Substrate-templated self-assembly of a catalyst leads to the formation of a high-energy structure. For detailed discussions, see refs (9−11).

A strong interest exists in the development of synthetic molecular devices and materials that operate out of equilibrium.^1−6^ In nature, nonequilibrium biological processes, such as directional motion of motor proteins, maintenance of concentration gradients, or the formation of high-energy structures, are all mediated by enzymes.^7^ Indeed, it has been shown that through catalysis, chemical energy stored in molecules with a high chemical potential can be exploited to drive chemical processes that are energetically uphill (Figure 1a).^8−11^ In the context of the development of dissipative materials, the substrate-templated self-assembly of catalytic structures has recently emerged as an attractive process because it inherently connects catalysis and self-organization.^12^ For example, this mechanism allows microtubules—the archetypical example of a biological dissipative structure^13^—to exploit chemical energy stored in GTP to carry out work.^14^ Recently, the first examples of synthetic structures that mimic the mechanism of microtubule formation—or essential parts of it—have been reported.^15−21^ In this process, substrate molecules activate building blocks for self-assembly (Figure 1b, i), which at the same time activates a catalytic pathway that converts the substrate into waste molecules with a lower templating ability (Figure 1b, ii). This step leads to the formation of the high-energy structure indicated in Figure 1a, which will spontaneously dissociate into the building blocks (Figure 1b, iii). Importantly, structural integrity of the high-energy structure can only be maintained as long as a continuous input of substrate is present. Yet, for application purposes, the complete loss of structural integrity of a dissipative material upon an interruption of the energy supply can pose a disadvantage. Here, we report a possible solution to that problem by describing a system that exploits two templated self-assembly processes. The first relies on a robust template for the self-assembly of surfactants in thermodynamically stable assemblies. The presence of these assemblies creates the conditions for a second self-assembly process templated by the substrate, which is—under the experimental conditions—thermodynamically disfavored in the absence of the strong template. The second self-assembly process leads to an insertion of additional surfactants in the assembly, which spontaneously form catalytic hotspots that cleave the substrate. Therefore, this system displays the substrate-induced formation of catalytic sites—essential characteristic of a dissipative structure, but this process is decoupled from the long-term stability of the structure which is guaranteed by the thermodynamically stable strong template. From an applicative point of view, an additional interesting feature is that the system is catalytically active at catalyst concentrations at which poor catalysis is observed in the absence of a robust template. This opens up new avenues for the development of catalytic and sensing systems with improved sensitivity.

## Results and Discussion

### Accelerated Catalysis in the Presence of ATP

**Figure 2:**
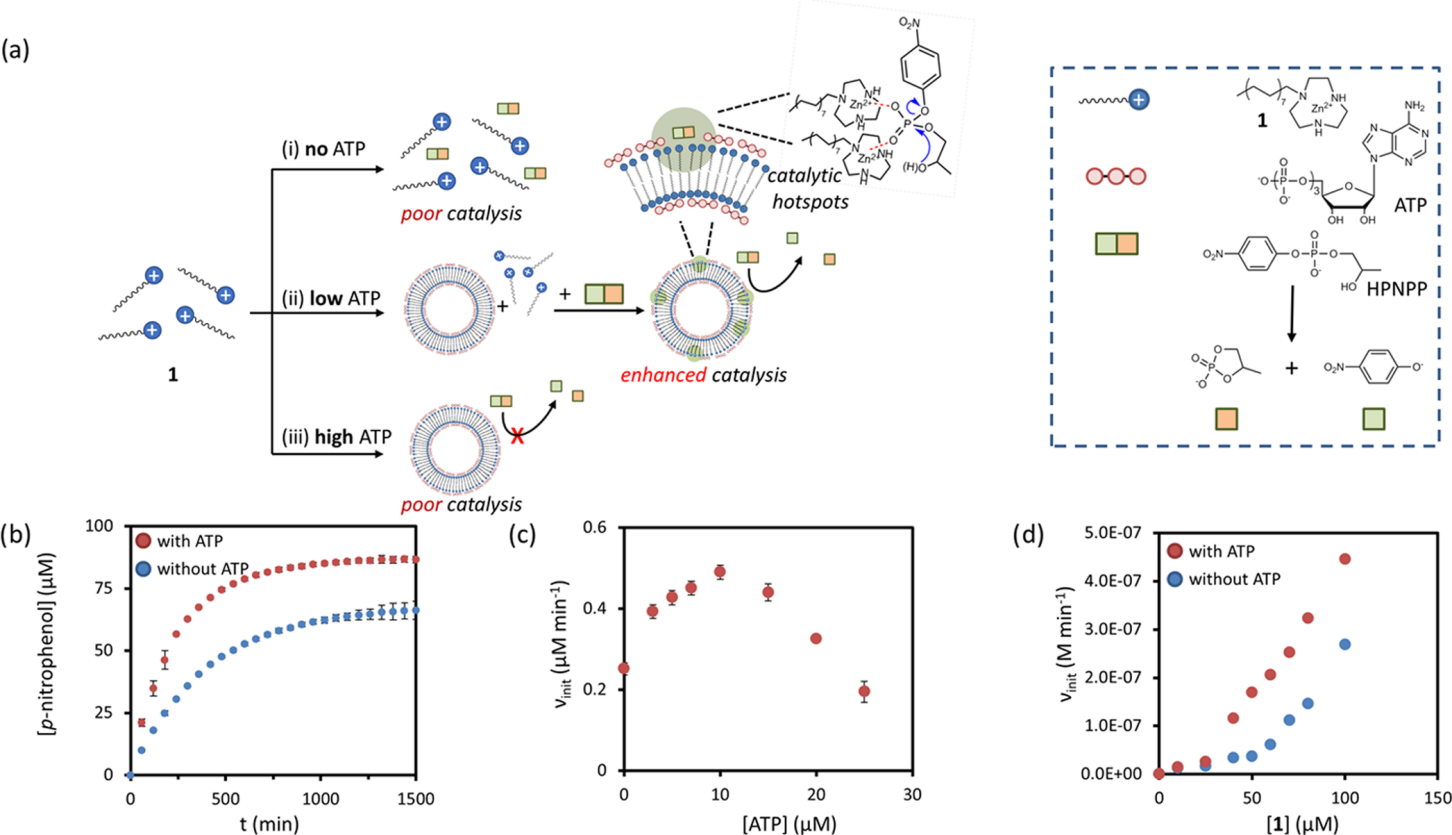
(a) Schematic representation of the effect of ATP concentration on the transphosphorylation of HPNPP catalyzed by assembled **1**: (i) in the absence of ATP, low catalytic activity is observed, (ii) low amounts of ATP lead to enhanced catalysis, and (iii) high amounts of ATP causes inhibition. (b) Concentration of the product *p*-nitrophenol as a function of time for a reaction mixture composed of **1** (100 μM) and HPNPP (200 μM) in the presence (red) and absence (blue) of ATP (5 μM). (c) Initial rate of the transphosphorylation of HPNPP as a function of the concentration of ATP present in the reaction mixture composed of **1** (100 μM) and HPNPP (200 μM). (d) Initial rate of the transphosphorylation of HPNPP (200 μM) as a function of the concentration of **1** in the presence (red) and absence (blue) of ATP (5 μM). Experimental conditions for (b–d): [HEPES] = 5 mM, pH = 7.0, *T* = 25 °C.

Previously, we have shown that the substrate 2-hydroxypropyl *p*-nitrophenyl phosphate (HPNPP) templates the self-assembly of surfactant C_16_TACN·Zn^2+^ (**1**, TACN = 1,4,7-triazacyclononane) into small spherical assemblies.^15^ The presence of HPNPP lowered the critical aggregation concentration (*cac*) of **1**, but high concentrations of substrate were required. Importantly, we observed that assembly formation activates the catalytic cleavage of HPNPP through the cooperative action of neighboring TACN·Zn^2+^-complexes in the assembly.^22,23^ The coincidence of substrate-templated assembly with the onset of catalytic activity implies that the system belongs to the class of substrate-templated catalysts discussed in the introduction. However, compared to a molecule such as ATP with multiple phosphate groups, HPNPP has a relatively weak affinity, and at low surfactant concentrations, no templated self-assembly of **1** takes place (Figure 2a, i). While studying the properties of this system in more detail, we made the surprising observation that the presence of a small amount of ATP (5 μM) in the reaction mixture led to increased catalytic performance both in terms of rate and turnover (Figures 2a, ii, 2b, and S1). This was not anticipated because the higher affinity of ATP for **1** compared to HPNPP was expected to lead to inhibition of the reaction rather than acceleration (Figure 2a, iii). Previously, such inhibition of catalysis was indeed observed when ATP was added to catalytic gold nanoparticles passivated with thiols containing the same catalytic TACN·Zn^2+^-head group (see Supporting Information, Section 4).^24^ It is of relevance for the studies discussed below that the gold nanoparticle data (Figure S2) show that catalysis was completely inhibited when around 25 μM ATP was added to a solution containing TACN·Zn^2+^-head groups at 100 μM and HPNPP at 200 μM. This shows that HPNPP cannot compete with ATP for binding at these concentrations, which is confirmed by the relative binding constants reported in Table S1.

The remarkable observation that the presence of ATP accelerated catalysis when surfactant **1** was used instead of gold nanoparticles initiated the studies described herein.

Two key experiments confirmed the positive effect of ATP on the catalytic activity of the assemblies. In the first experiment, a constant concentration of **1** (100 μM) was incubated with different concentrations of ATP (0–25 μM), after which HPNPP (200 μM) was added. We found that assembly formation led to some turbidity and therefore introduced a lag time of 15 min between ATP and HPNPP addition to avoid that the initial changes in absorbance caused by turbidity would affect the initial rate determination. A control experiment in which ATP and HPNPP were added simultaneously gave the same rate, indicating that the lag time did not affect the results (Figure S5). The catalytic activity was followed by measuring the increase in absorbance at 405 nm originating from the release of *p*-nitrophenol (Figure S3). A plot of the initial rate as a function of the concentration of ATP gave a bell-shaped curve, confirming indeed an accelerating effect on catalysis for small amounts of ATP, but, on the other hand, an inhibitory effect when larger amounts of ATP were present (Figure 2c).

It should be noted that the experiment reported in Figure 2c was carried out at a 100 μM concentration of **1**. Because this concentration is above the *cac* for **1** in the presence of 200 μM HPNPP (∼40 μM, Figure S8), this implies that some HPNPP-templated assemblies are present in the system. This is indeed reflected by the observation of some catalytic activity in the absence of ATP. The situation is therefore different than that represented in Figure 2a, which suggests that no assemblies are present in the absence of ATP. To ensure that the presence of HPNPP-templated assemblies does not affect the observed rate acceleration induced by ATP, we repeated the experiment shown in Figure 2c at reduced concentrations of **1** (50 and 20 μM). Previously, we have shown that also at these lower concentrations of **1**, ATP is able to template assembly formation.^25,26^ At both concentrations, we see a bell-shaped curved when the initial rate was plotted as a function of the concentration of ATP (Figure S4). This shows that for the accelerating effect by ATP it is irrelevant whether **1** is unassembled or part of an HPNPP-templated assembly.

In the second experiment, the catalytic activity was measured in mixtures containing increasing concentrations of **1** (0–100 μM) at a constant concentration of HPNPP (200 μM) in the presence and absence of ATP (5 μM) (Figures 2d and S6). In the absence of ATP, catalytic activity started to increase when **1** reached a concentration of around 50 μM, which is close to the *cac* of **1** in the presence of 200 μM HPNPP (Figure S8). However, in the presence of ATP, catalytic activity started to increase at a significantly lower concentration of **1** of just around 25 μM. This is an important observation because it shows that the presence of ATP causes the system to be catalytically active at lower concentrations of the catalyst (*i.e.*, surfactant **1**).

### Structural Studies

**Figure 3:**
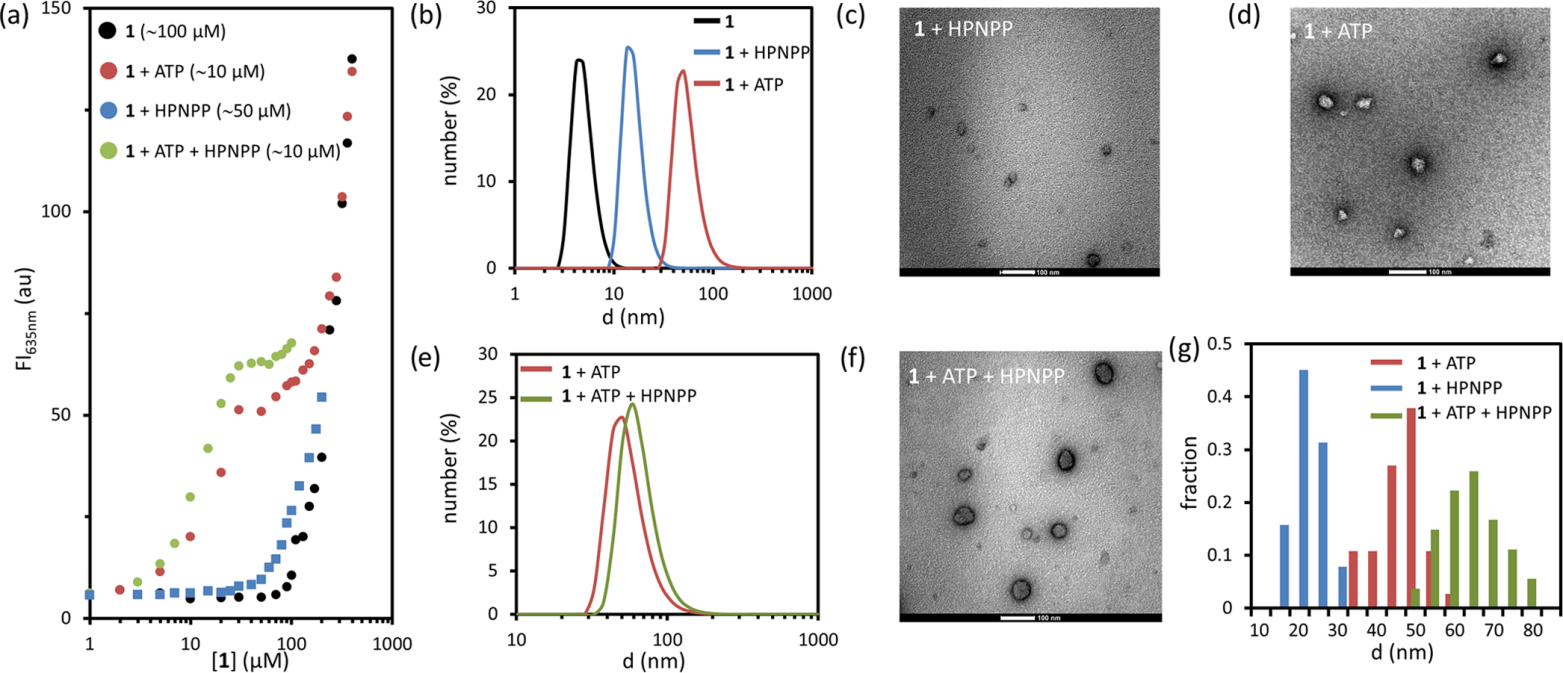
(a) Fluorescence intensity from Nile Red at 635 nm as a function of the concentration of **1** present in mixtures containing: no other components (black), ATP (red), HPNPP (blue), ATP and HPNPP (green). Critical aggregation concentrations for each sample are reported between parenthesis in the legend. Experimental conditions for 3a: [**1**] = 100 μM, [ATP] = 10 μM, [HPNPP] = 100 μM, [Nile Red]= 2 μM, [HEPES] = 5 mM, pH = 7.0, T = 25 °C. (b) DLS (number %) of solutions containing: **1** (black), **1** and HPNPP (blue), **1** and ATP (red). (c) TEM image of a solution containing **1** and HPNPP. (d) TEM image of a solution containing **1** and ATP. (e) DLS (number %) of solutions containing: **1** and ATP (red), **1**, ATP, and HPNPP (green). (f) TEM image of a solution containing **1**, ATP, and HPNPP. (g) Size distribution of structures (around 40–50) observed in TEM images of solutions containing **1** + ATP (red), 1 + HPNPP (blue), and **1** + ATP + HPNPP (green). Experimental conditions for 3b–g: [**1**] = 100 μM, [ATP] = 10 μM, [HPNPP] = 200 μM, [HEPES] = 5 mM, pH = 7.0, *T* = 25 °C.

To understand the origin of the accelerating effect of low amounts of ATP on catalysis, we investigated the templated self-assembly process in more detail. To have a reference point, we first studied the templating capacities of ATP and HPNPP on their own. Determination of the *cac* using Nile Red as a hydrophobic fluorescent probe for assembly formation revealed that assemblies formed at much lower concentrations in the presence of ATP (10 μM, *cac* ≈ 10 μM) compared to HPNPP (100 μM, *cac* ≈ 50 μM, 200 μM, *cac* ≈ 40 μM) (Figures 3a and S8). This confirmed our previous studies, which have already shown that ATP is a more effective template compared to HPNPP.^15,25,26^ Dynamic light scattering (DLS) and transmission electron microscopy (TEM) measurements revealed a larger size for ATP- *vs* HPNPP-templated assemblies (*d*_ATP_ ≈ 50 nm and *d*_HPNPP_ ≈ 20 nm, respectively) (Figure 3b–d).

Next, we examined how the ATP-templated self-assembly of **1** was affected by the presence of HPNPP. Repetition of the fluorescence titration experiments with Nile Red revealed that the concurrent presence of both ATP (10 μM) and HPNPP (100 μM) had no effect on the c*ac*, which remained around 10 μM but led to higher fluorescence intensity (Figure 3a, green). The latter observation suggests that in the presence of HPNPP, more apolar domain is available in the system for the uptake of Nile Red compared to the system in which just ATP is present (Figure 3a). Additional fluorescence titration experiments showed that the difference in fluorescence intensity in the absence and presence of HPNPP increased at higher HPNPP concentrations (Figure S7). Structural changes in the ATP-templated assemblies in the presence of HPNPP emerged from DLS and TEM measurements. The concurrent presence of both ATP and HPNPP led to an increase in the hydrodynamic diameter of around 10 nm (*d*_ATP_ ≈ 50 nm; *d*_ATP/HPNPP_ ≈ 60 nm) (Figures 3e and S9). The size increase was confirmed by a statistical analysis of the structures visible in the TEM images (Figures 3f+g, S10, and S11).

### Catalytic Hotspots

**Figure 4:**
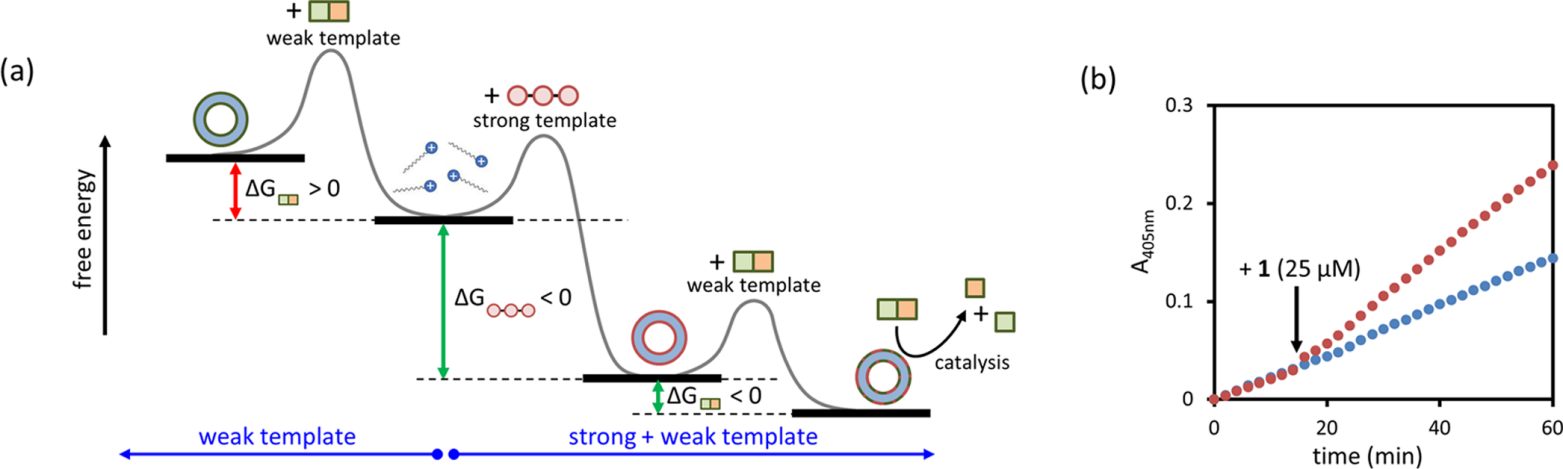
(a) Thermodynamically controlled self-assembly process induced by a strong template makes a second self-assembly process induced by a weak template thermodynamically favorable under conditions at which the latter process would not occur by itself. (b) Absorbance at 405 nm as a function of time for solutions containing **1** (100 μM), ATP (25 μM), and HPNPP (200 μM). To one of the solutions (red), additional **1** (25 μM) was added after *t* = 15 min; the other solution (blue) was left unaltered.

Based on these observations, we postulate that the addition of HPNPP to a solution of ATP-templated assemblies induces the insertion of additional free surfactant **1** in the assemblies, leading to the formation of catalytic hotspots. We refer to catalytic hotspots as small clusters of surfactant **1** in ATP-templated assemblies that catalyze HPNPP through cooperative action between neighboring TACN·Zn^2+^-head groups (Figure 2a).^22,23^ This would explain the increased availability of hydrophobic domains in the system when HPNPP is present in the system and the larger assembly size. There are two intriguing features to this dual self-assembly process (Figure 4). First, HPNPP acts as a template for the self-assembly of catalytic clusters of **1** at a surfactant concentration at which it is unable to template assembly formation in the absence of ATP. In other words, the presence of the high-affinity template ATP favors templated self-assembly by the weak-affinity template HPNPP. This is attributed to the lower entropy cost for the HPNPP-templated insertion of **1** into preorganized ATP-templated assemblies as compared to the entropy cost associated with the formation of assemblies of **1** templated just by HPNPP. The second feature is that the formation of catalytic hotspots has the important consequence that catalysis occurs at concentrations of **1** for which no catalytic activity is observed in the absence of ATP (Figure 2d).

Our hypothesis implies that the possibility of forming catalytic hotspots critically depends on the ratio of **1**, ATP, and HPNPP in the system. Previously, we have shown that ATP-templated assemblies of **1** contain ATP and **1** in a 1:3 ratio,^25^ which is also confirmed by the maximum fluorescence intensity reached after 30 μM of **1** had been added to a 10 μM solution of ATP (Figure 3a). Catalytic hotspots can form when the amount of ATP is below that ratio so that excess **1** is available in the system. This prerequisite explains the bell-shaped curves reported in Figure 2c and Figure S4. At low concentrations of ATP, ATP-templated assemblies of **1** form, but additional **1** is still abundantly available for the formation of catalytic hotspots upon the addition of HPNPP. As the concentration of ATP reaches the 1:3 ratio with respect to **1**, surfactant **1** becomes progressively involved in binding to ATP and is no longer available for the formation of catalytic hotspots. Considering the much higher affinity of ATP for **1** compared to HPNPP, the result is an inhibition of catalysis when ATP reaches the 1:3 ratio. In coherence with this explanation, we observed an immediate increase in catalytic activity when an additional batch of **1** was added to a mixture containing **1** and a relatively high concentration of ATP (25 μM) at which catalytic activity was low (Figure 4b).

### FRET Experiments

**Figure 5:**
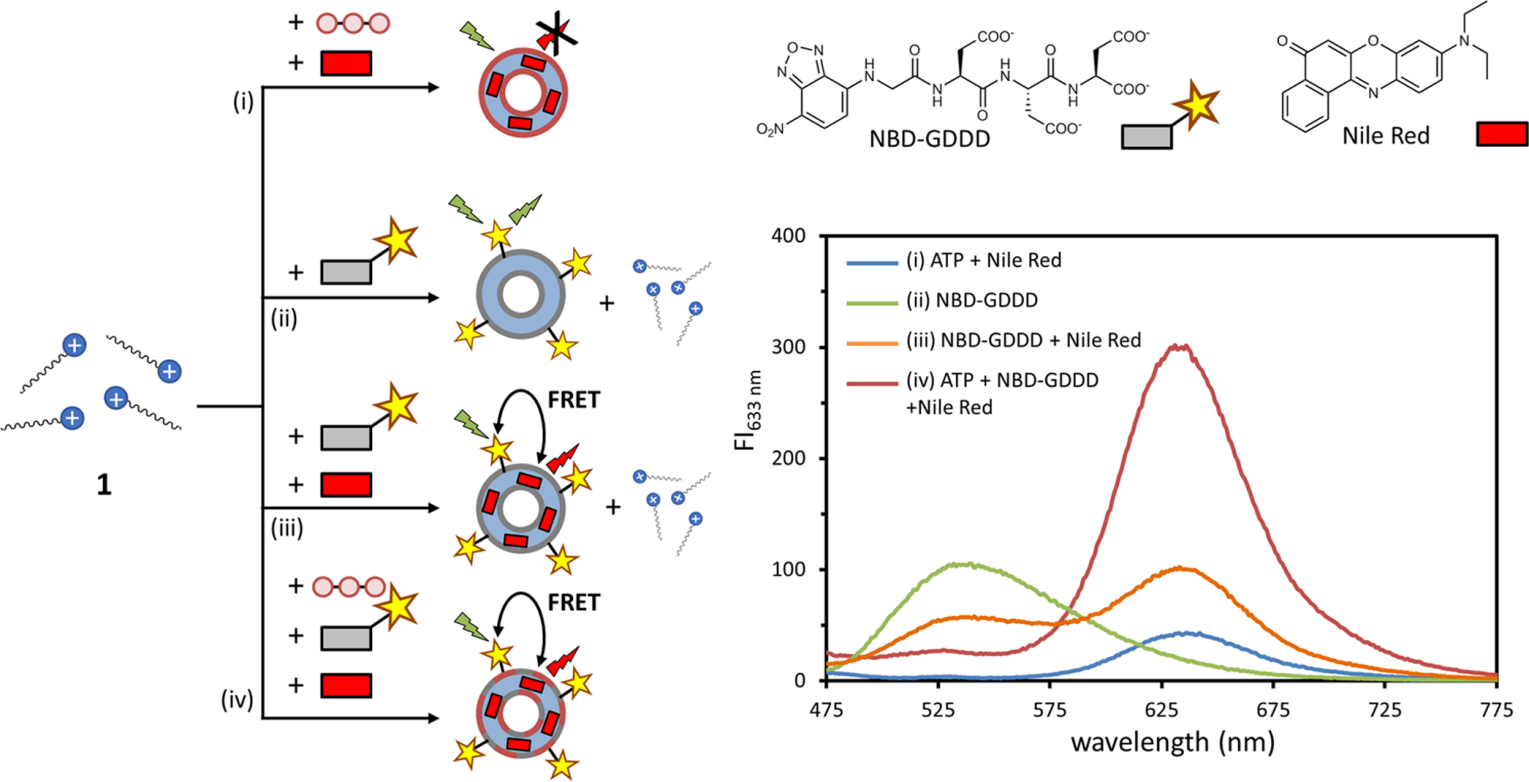
Schematic representation of the samples studied by fluorescence to demonstrate the coexistence of NBD-GDDD and ATP in the same assembly. Sample compositions: (i) **1** (100 μM), ATP (10 μM), and Nile red (2 μM); (ii) **1** (100 μM), NBD-GDDD (3 μM)—a small amount of NBD-GDDD was added to ensure the presence of unassembled **1** in the system. (iii) **1** (100 μM), NBD-GDDD (3 μM), Nile red (2 μM), (iv) **1** (100 μM), ATP (10 μM), NBD-GDDD (3 μM), and Nile red (2 μM). In samples (ii–iv), a small amount of NBD-GDDD was added to ensure the presence of unassembled **1** in the system.

Our explanation for the increase in catalytic activity upon the addition of ATP relies on the stabilizing effect of ATP-templated assemblies on the formation of catalytic hotspots between HPNPP and additional **1**. This implies that ATP and HPNPP must coexist in the same assembly. To lend support for this hypothesis, we looked for additional proof that could support the coexistence of a strong and weak template in the same assembly. In previous work, we had studied the interaction between gold nanoparticles passivated with thiols containing the same TACN·Zn^2+^-head groups (Section 4 of the Supporting Information) and a negatively charged peptide NBD-GDDD equipped with the fluorophore NBD (7-nitro-2,1,3-benzoxadiazole).^27^ We have shown that the NBD-GDDD probe has a significantly lower affinity compared to ATP, which can be attributed to the presence of carboxylate- rather than phosphate moieties.^28^ Because NBD (λ_ex_ = 450 nm, λ_em_ = 550 nm) forms a FRET-couple with Nile Red (λ_ex_ = 570 nm, λ_em_ = 633 nm), we argued that we could use FRET to trace the location of the weaker template NBD-GDDD in the system (Figure S14). Therefore, we prepared a series of samples and measured the fluorescence emission spectra exciting at the absorption maximum of NBD-GDDD (450 nm) (Figure 5). A low fluorescence emission from Nile Red at 633 nm was observed when just ATP (10 μM) was added to a solution of **1** (100 μM) and Nile Red (2 μM) because Nile Red absorbs poorly at 450 nm (Figure 5, i). On the other hand, addition of just a very small amount of NBD-GDDD (3 μM) to a solution of **1** (100 μM) resulted, as expected, in a strong fluorescence emission originating from NBD (λ_max_ = 450 nm) (Figure 5, ii). Addition of Nile Red to that sample resulted in a decrease in the fluorescence intensity at 450 nm but an increase at 633 nm (Figure 5, iii). This is attributed to FRET between NBD bound to the assembly surface and Nile Red present in the hydrophobic domain of the assemblies. Importantly, the additional presence of ATP (10 μM) in the same sample caused a strongly increased FRET, evidenced by a nearly complete disappearance of the emission at 450 nm (NBD) and a strong increase at 633 nm (Nile Red) (Figure 5, iv). The enhanced FRET in this final system indicates the coexistence of ATP and NBD-GDDD in the templated assemblies. If NBD-GDDD and ATP would have resided on different assemblies, the addition of ATP would only have marginally affected the fluorescence emission of Nile Red.

### FCS Measurements

**Figure 6:**
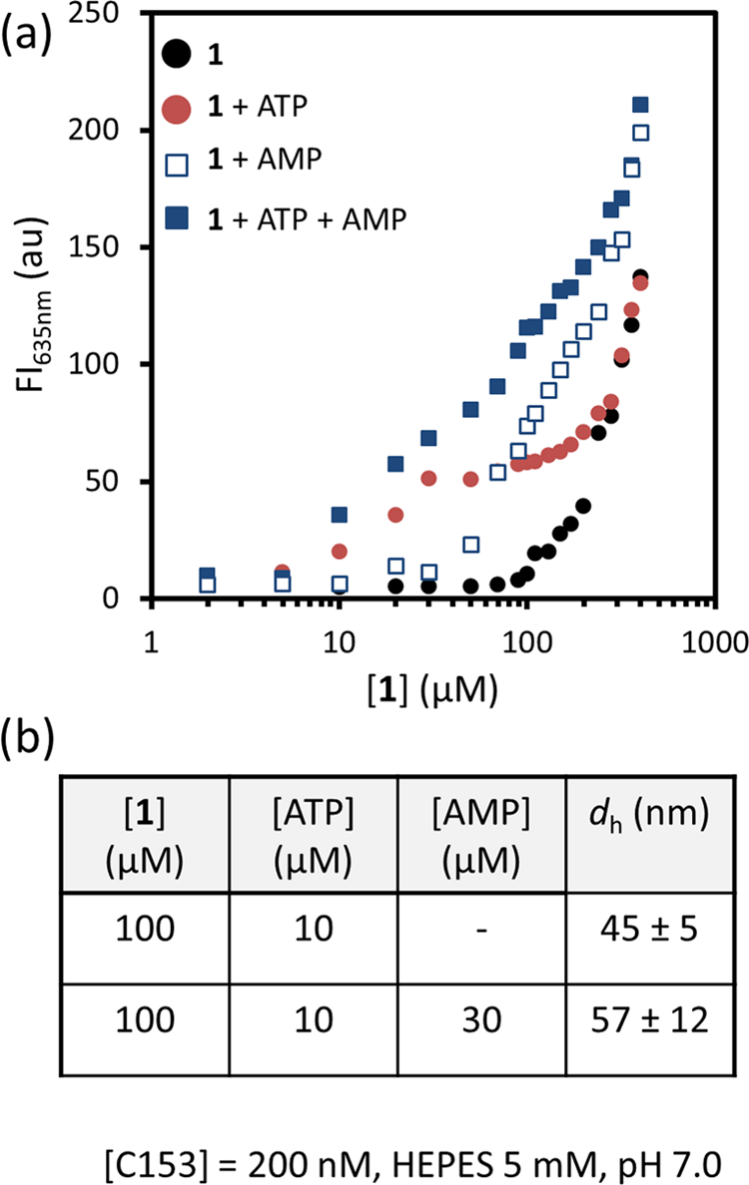
(a) Fluorescence intensity from Nile Red at 635 nm as a function of the concentration of **1** present in mixtures containing: no other components (black), ATP (red, 10 μM), AMP (open blue squares, 30 μM), ATP (10 μM) and AMP (30 μM) (closed blue squares). (b) Assembly size (diameter in nm) as determined by FCS measurements of two solutions containing **1**, ATP, and AMP in the indicated concentrations. Experimental conditions: [HEPES] = 5 mM, pH = 7.0, *T* = 25 °C.

Finally, to investigate the generality of the dual-templated self-assembly process involving the simultaneous use of a strong and weak affinity template, we carried out additional studies using adenosine monophosphate (AMP) rather than HPNPP in combination with ATP. Previous studies have shown that, like HPNPP, AMP has a much lower affinity compared to ATP for multivalent surfaces containing the TACN·Zn^2+^ - complex.^24,25^ Observation of facilitated assembly of **1** templated by AMP in the presence of ATP would suggest that the enhanced templating ability of the weak template in the presence of the strong template may be a general phenomenon.^29,30^ The stability of AMP and the absence of spectral overlap with the fluorogenic probe C153 permitted the use of fluorescence correlation spectroscopy (FCS) as an alternative technique to determine the assembly size. FCS relies on the measurement of the diffusion coefficient of the apolar fluorophore C153 entrapped in the hydrophobic domain of the assemblies. Fluorescent titration experiments confirmed that AMP on its own is a modest template and reduced the *cac* of **1** to around 50 μM when present at a 30 μM concentration (Figure 6a, open blue squares; see also Figure S8). Importantly, repetition of the titration experiment in the concurrent presence of ATP (10 μM) and AMP (30 μM) gave the same result as observed for HPNPP: the increase in fluorescence intensity started at a concentration of **1** of 10 μM (corresponding to the *cac* in the presence of ATP), but higher fluorescent intensities were measured at each concentration of **1** (Figure 6a, closed blue squares). FCS measurements confirmed that larger assemblies formed when AMP was present together with ATP. Measurements were conducted at a concentration of **1** equal to 100 μM and ATP (10 μM), both in the presence and absence of AMP (30 μM). The presence of AMP resulted in larger assemblies with an increase in diameter corresponding to around 10 nm (Figure 6b). This observation was confirmed by DLS (Figure S9), suggesting indeed a general applicability of the dual self-assembly processes involving a strong and weak template.

## Conclusions

In conclusion, we have developed a system that combines a thermodynamic and dissipative self-assembly process. The presence of the ATP-templated assemblies generates the conditions for the activation of the dissipative self-assembly process in which the substrate HPNPP templates the formation of catalytic hotspots for its own destruction. The facilitated substrate-induced assembly of additional building blocks at low concentrations is attributed to the preorganization effect exerted by the ATP-templated assembly. Within the context of the development of dissipative structures, the system represents important features. The decoupling of long-term stability of the structure—guaranteed by the thermodynamically controlled self-assembly process—from the energy dissipation process avoids the necessity for a continuous supply of energy to maintain structural stability. In addition, the fact that catalysis occurs at lower building block concentrations implies that the system is better attributed to harvest the chemical potential of the substrate to carry out work. Regrettably, the current system does not allow us to demonstrate these features because of waste interference. That is, previously, we have shown that the waste products of HPNPP cleavage have the same templating ability as the substrate.^15^ This implies that after conversion of the substrate into waste, the assemblies reside in a thermodynamically stable state stabilized by waste. This makes the system irresponsive toward new fuel additions. From an applicative point of view, an important feature of the system is the observation that the hybrid system displays catalysis at lower catalyst concentrations. This phenomenon may be of use for developing catalytic and sensing systems with improved sensitivity.

## Supporting Information Available

Materials and instrumentation, experimental procedures, and additional supporting data (PDF)
