# Supplementary material for: Formation of Catalytic Hotspots in ATP-Templated Assemblies
Source: J Am Chem Soc. 2022 Dec 28;145(2):898–904. doi: 10.1021/jacs.2c09343 (PMC9853849; doi:10.1021/jacs.2c09343)
Supplement: Supplementary file 1 — ja2c09343_si_001.pdf [file ja2c09343_si_001.pdf]

Supporting Information

Formation of Catalytic Hotspots in ATP-Templated Assemblies

Krishnendu Das, Haridas Kar, Rui Chen, Ilaria Fortunati, Camilla Ferrante, Paolo Scrimin, Luca Gabrielli, Leonard J. Prins*

Department of Chemical Sciences, University of Padova, Via Marzolo 1, 35131 Padova, Italy

Table of Contents

Item	page
1. Materials and instrumentation	2
2. Data Analysis	4
3. HPNPP catalysis by surfactant **1** in the absence and presence of ATP	5
4. HPNPP catalysis by AuNP·Zn^{2+} in the presence of increasing concentration of ATP	6
5. HPNPP catalysis by **1** in the presence of increasing concentrations of ATP	7
6. Effect of ATP on the onset concentration of **1** for HPNPP catalysis	10
7. Fluorescence titrations of increasing amounts of **1** to solutions containing a fixed amount of ATP in the presence and absence of HPNPP	11
8. DLS studies of the ATP-HPNPP system	13
9. TEM analysis	14
10. In-situ acceleration of HPNPP catalysis by adding **1**	15
11. DLS study to explore the templating ability of AMP	16
12. DLS study of the ATP-AMP system	17
13. FRET experiments	18
14. Supplemental References	19

1. Materials and instrumentation

Materials:

All commercially available reagents were purchased from Sigma Aldrich and used as received. 4-(2-Hydroxyethyl)-1-piperazineethanesulfonic acid (HEPES) was purchased from Sigma-Aldrich and used without further purification. Fluorophores Nile Red and coumarin153 (C153) were procured from Sigma-Aldrich and were used without further purification.

$Zn(NO_3)_2$ was analytical grade product and the concentration of the stock solution was determined by ICP (inductively coupled plasma) analysis.

Adenosine 5'-triphosphate (ATP) disodium salt hydrate, and adenosine 5'-monophosphate (AMP) sodium salt stock solutions were prepared in MilliQ water by weight and the exact concentration was calculated by UV-Vis spectroscopy using the molar extinction coefficient: ε_{259} (ATP, AMP) = 15400 M^{-1} cm^{-1}.

The synthesis and characterization of surfactant **1** and HPNPP have already been reported.[1] The stock solutions of **1** (without Zn^{2+}) and HPNPP were prepared by weight in MilliQ water to give a concentration of 5 mM.

Gold nanoparticles AuNP.Zn^{2+} passivated with thiols containing the same catalytic TACN·Zn^{2+}-head group were synthesized following a reported protocol.[2] The head group concentration of AuNP AuNP.Zn^{2+} was determined from kinetic titrations using ATP_f as reported previously.[3]

Instruments

pH Measurements: The pH of buffer solutions was determined at room temperature using a Metrohm 632 pH meter equipped with a Ag/AgCl/KCl reference electrode and calibrated with standard buffer solutions at pH 7.00.

UV-Vis Measurements: UV-Vis spectra and kinetics were recorded on a Varian Cary50 spectrophotometer equipped with thermostatted multiple cell holders.

Fluorescence Spectroscopy: Fluorescence measurements were recorded on Varian Cary Eclipse fluorescence spectrophotometer equipped with a thermostatted cell holder.

TEM Analysis: TEM images were recorded on a Jeol 300 PX electron microscope. One drop of sample was placed on the sample grid for 1 minute. For staining purposes a drop of uranyl acetate (2%) was added and the sample was left for 30 s. The solvent was evaporated before the stained grid was imaged.

FCS Analysis: Fluorescence correlation spectroscopy, the emission signal was sent to two single-photon counting avalanche photodiodes (SPAD, MPD, Italy) and registered with PicoHarp 300 TCSPC electronics (PicoQuant). The fitting of exponential decay curve and FCS data were performed with the Symphotime software (PicoQuant).

2. Data analysis

UV-vis kinetic data:

All the UV-vis kinetics were recorded using scanning kinetic mode where entire spectra were recorded over increasing time intervals. The data were converted to kinetic profiles by plotting the absorbance at 405 nm (the absorption of the deprotonated form of hydrolytic product *p*-nitrophenol) against time. The absorbance data were corrected by subtracting the initial absorbance (at t = 0 min) from the rest of the absorption values to nullify the initial background signal. The absorbance data were converted to concentration by using the molar extinction coefficient for *p*-nitrophenol at pH 7.00 (10800 M^{-1} cm^{-1}).

Fluorescence data:

All the fluorescence experiments were carried out by exciting the fluorophore Nile red (2 μM) at 570 nm and recording the corresponding emission intensity at 635 nm. The excitation and emission slit width were set to 5 and 10 nm, respectively.

3. HPNPP catalysis by surfactant 1 in the absence and presence of ATP

A 100 μM solution (final concentration) of surfactant **1** was prepared in two separate cuvettes using an aqueous solution of HEPES buffer (5 mM, pH 7.0) as the solvent. To one of the cuvettes 5 μM ATP was added and the solution was left for 15 minutes. Just before starting the kinetics 200 μM HPNPP was added to both cuvettes. The data were recorded using scanning mode by monitoring the change in the absorption spectra (300-500 nm) as a function of time (Figure S1). The increasing absorption at 405 nm originates from the formation of the chromogenic product *p*-nitrophenol (deprotonated). The kinetic profile reported in Figure 2b of the manuscript was obtained by plotting the absorption at 405 nm against time. The absorbance data were corrected by subtracting the initial absorbance (at t = 0 min) from the rest of the absorption values to correct for the initial background absorbance. The experiment was carried out in duplo.

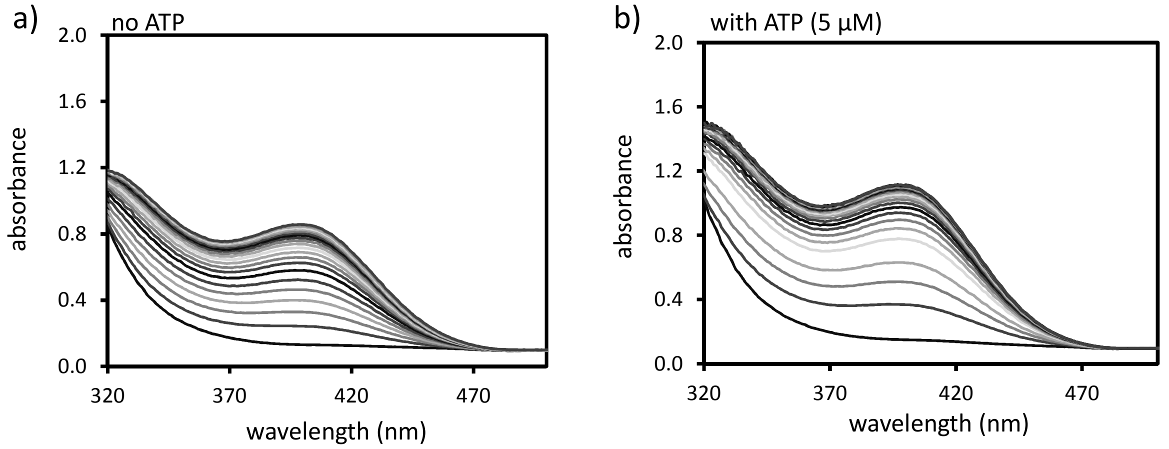

Figure S1. Kinetic traces of HPNPP catalysis by **1** in (a) the absence and (b) the presence of ATP. The spectra were recorded in scanning kinetic mode. [**1**] = 100 μM, [HPNPP] = 200 μM, [ATP] = 5 μM, HEPES buffer (5 mM, pH 7.0), 25 °C.

4. HPNPP catalysis by AuNP·Zn^{2+} in the presence of increasing concentration of ATP

The synthesis and characterization of AuNP·Zn^{2+} (Figure S2a) has been reported before.[2,3,4] A solution of AuNP·Zn^{2+} (final concentration of 100 μM referring to the head group concentration) was prepared in each of 9 different cuvettes using an aqueous solution of HEPES buffer (5 mM, pH 7.0) as the solvent. To 8 cuvettes different concentrations of ATP (3-30 μM) were added (Figure S2b). One cuvette was left only with AuNP·Zn^{2+} as reference. Just before starting the kinetic measurements 200 μM HPNPP was added to each of the cuvettes and the absorption was recorded using kinetic mode at 405 nm. The absorbance data were corrected by subtracting the initial absorbance (at t = 0 min) from the rest of the absorption values to nullify the initial background signal (Figure S2c). The absorbance data were converted to concentration by using the molar extinction coefficient 10800 M^{-1} cm^{-1}.Initial reaction rates were determined by using the slope of the linear part of the concentration vs time plot (Figure S2d).

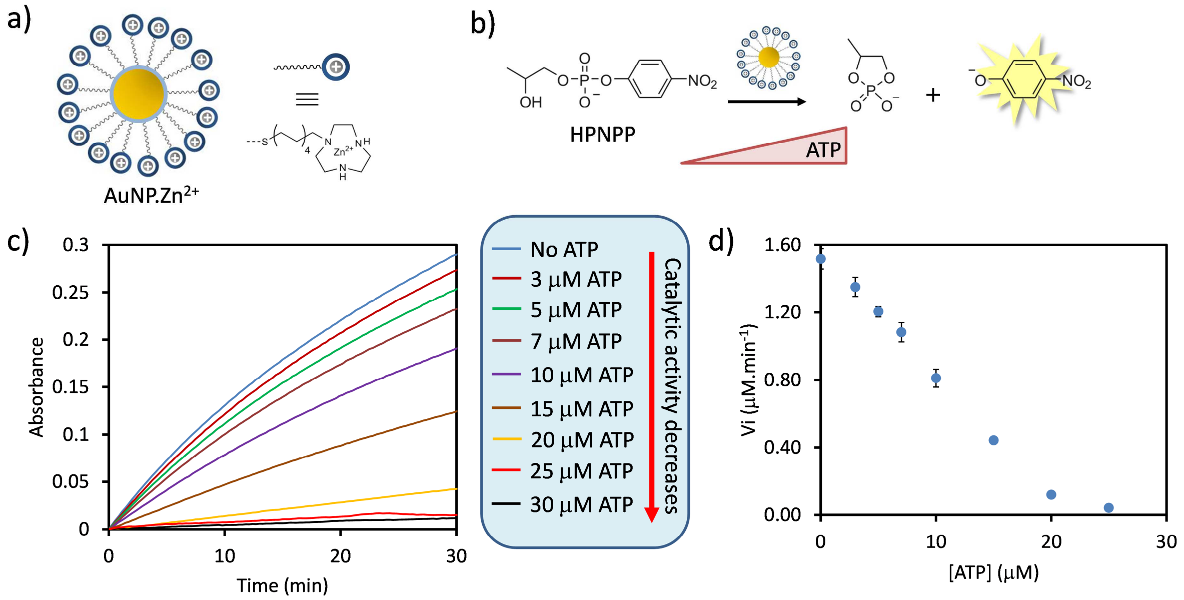

Figure S2. a) Structure of AuNP.Zn^{2+}. b) Schematic representation of the inhibition of catalysis by increasing amounts of ATP. c) Change in the absorbance at 405 nm over time at different concentrations of ATP. (d) Initial rate of the transphosphorylation of HPNPP as a function of the concentration of ATP. Experimental conditions: [AuNP.Zn^{2+}] = 100 μM, [HPNPP] = 200 μM, [ATP] = 0-30 μM, HEPES buffer (5 mM, pH 7.0), 25 °C.

Table S1. Previously reported dissociation constants of substrates and ligands for AuNP·Zn^{2+}

Compound	Kd (M)	Rel. Kd	Reference
HPNPP	5.8×10^{-4}	1	5
AMP	$2.0\text{x}10^{-5}$	29	6
ATP	$<5.0\text{x}10^{-7}$	>1000	6

5. HPNPP catalysis by 1 in the presence of increasing concentrations of ATP

A solution of surfactant **1** (100 μM final concentration) was prepared in each of 8 different cuvettes using an aqueous solution of HEPES buffer (5 mM, pH 7.0) as the solvent. To 7 cuvettes different concentrations of ATP (3-25 μM) were added. After addition of ATP the cuvettes were left for 15 minutes. One cuvette was left only with surfactant **1** as control. Just before starting the experiment 200 μM HPNPP was added to each of the cuvettes and data were recorded in scanning mode. The kinetic profiles were obtained by plotting the absorbance at 405 nm as a function of time (Figure S3). The absorbance data were corrected by subtracting the initial absorbance (at t = 0 min) from the rest of the absorption values to nullify the initial background signal. The absorbance data were converted to concentration by using the molar extinction coefficient 10800 M^{-1} cm^{-1}. Initial velocities corresponded to the slope of the linear part of the concentration vs time plot.

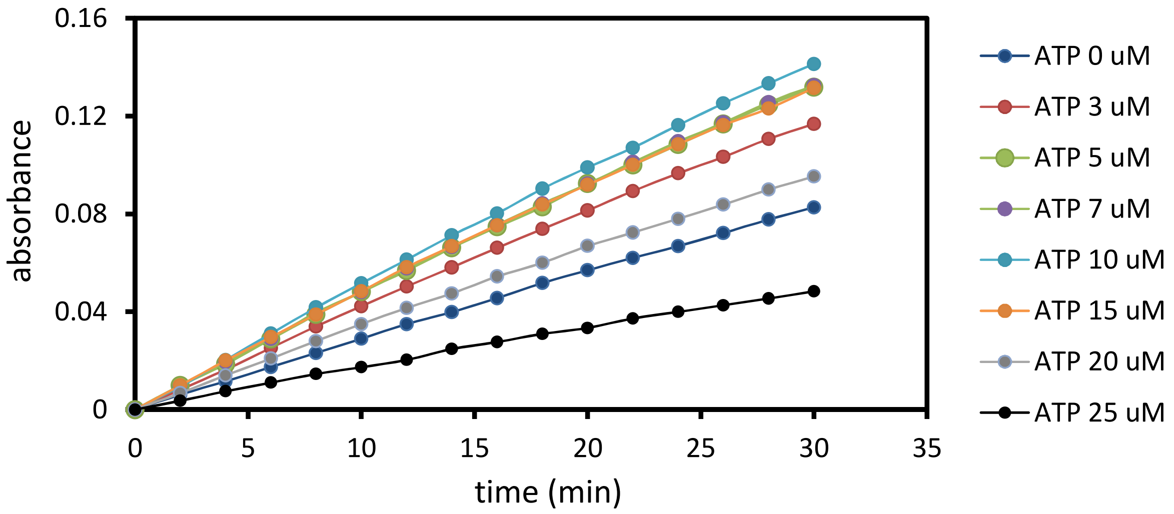

Figure S3. Change in the absorbance at 405 nm over time at different concentration of ATP. Lines are added to guide the eye. [**1**] = 100 μM, [HPNPP] = 200 μM, [ATP] = 0-25 μM, HEPES buffer (5 mM, pH 7), 25 °C.

The experiment was repeated at different final concentrations of **1** equal to 50 and 20 μM respectively. The plots of the initial rates as function of the concentration of ATP (analogous to Figure 2c) in the manuscript are given in Figure S4.

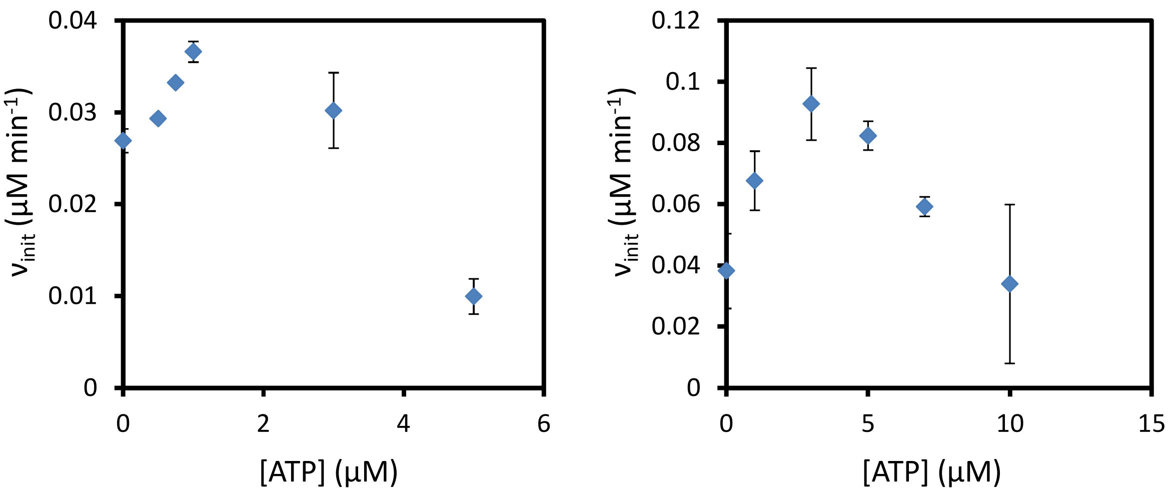

Figure S4. Initial rate of the transphosphorylation of HPNPP as a function of the concentration of ATP present in the reaction mixture composed of **1** (a: 20 μM; b: 50 μM) and HPNPP (200 μM). Experiments were carried out in duplo. Experimental conditions: [HEPES] = 5 mM, pH = 7.0, T = 25 °C.

As indicated in the procedures, experiments of this kind were typically carried out by waiting 15 minutes between the addition of ATP and HPNPP. To demonstrate that the lag time does not affect the observations we carried out an experiment in which ATP and HPNPP were added simultaneously. A solution of surfactant **1** ($C_{16}TACN{\cdot}Zn^{2+}$, 50 μM final concentration) was prepared in each of four different cuvettes using an aqueous solution of HEPES buffer (5 mM, pH 7.0) as the solvent. In one series (2 cuvettes), 3 μM ATP and 200 μM HPNPP were added together. In the other series (2 cuvettes), 3 μM ATP was first added and the solution was left for 15 minutes before 200 μM HPNPP was added. The data were recorded using scanning mode by monitoring the change in the absorption spectra (250-600 nm) as a function of time. The kinetic profiles were obtained by plotting the absorbance at 405 nm as a function of time. The absorbance data were corrected by subtracting the initial absorbance (at t = 0 min) from the rest of the absorption values to nullify the initial background signal. A plot of absorbance vs time displays no difference between the samples in which ATP and HPNPP were added simultaneously and the samples in which HPNPP was added 15 minutes after ATP (Figure S5).

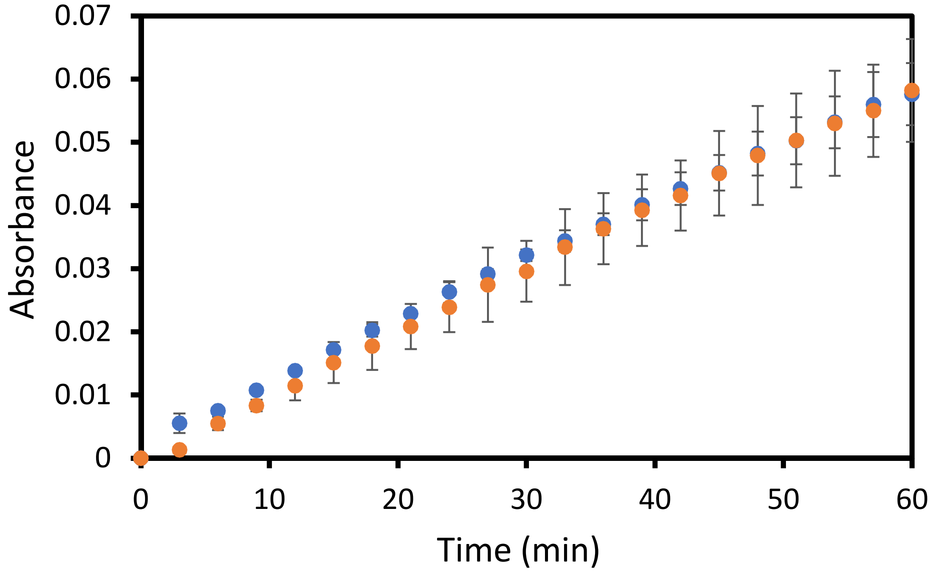

Figure S5. Increase in absorbance at 405 nm as a function of time when to a solution of **1** were added: (i) ATP and HPNPP simultaneously (blue); (ii) ATP first and HPNPP after 15 minutes (orange). Experiments were carried out in duplo. Experimental conditions: [**1**] = 50 µM, [ATP]= 3 µM, [HPNPP]= 200 µM, HEPES buffer (pH 7, 5 mM), T=25°C.

6. Effect of ATP on the onset concentration of 1 for HPNPP catalysis

The effect of ATP on the onset concentration of **1** for HPNPP catalysis was investigated by measuring the change in initial velocity of HPNPP catalysis as a function of surfactant **1** concentration in the presence and absence of ATP in aqueous buffer solution (5 mM HEPES, pH 7.0). Two series of cuvettes were prepared. In one series 200 μM HPNPP was added to each cuvette and in the other series along with 200 μM HPNPP also additional 5 μM ATP was added to each cuvette. Afterwards, different amounts of surfactant **1** were added to each of the cuvettes and initial velocities of HPNPP catalysis were measured. A plot of initial velocity vs surfactant concentration clearly displays the positive effect of the presence of ATP on the reaction rate (Figure 2d, manuscript).

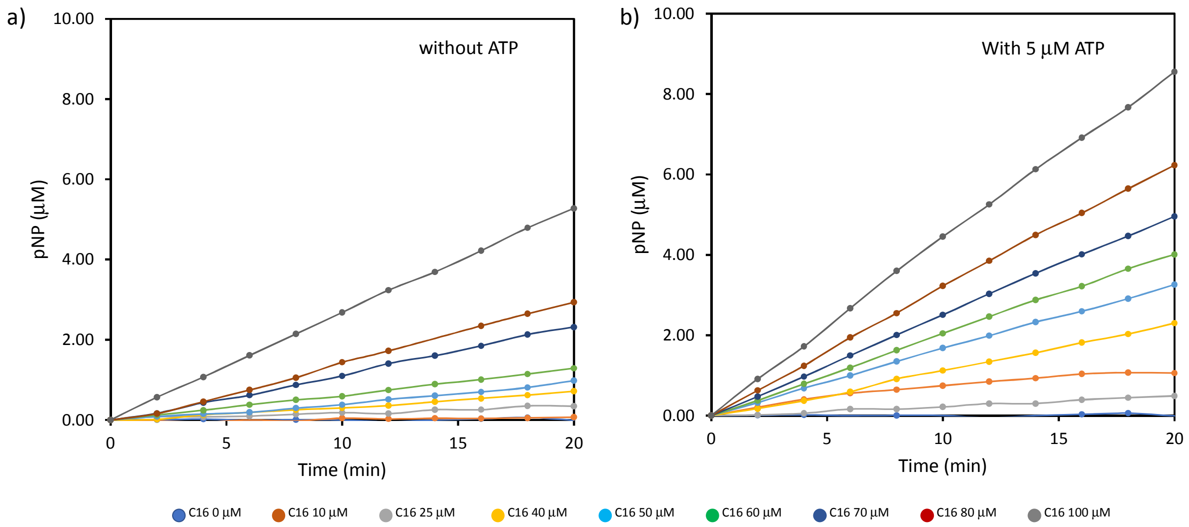

Figure S6. Kinetic profile of HPNPP catalysis as a function of increasing concentration of **1** in (a) the absence and (b) the presence of ATP. Lines are added to guide the eye. [**1**] = 0-100 μM, [HPNPP] = 200 μM, [ATP] = 5 μM, HEPES buffer (5 mM, pH 7), 25 °C.

7. Fluorescence titrations of increasing amounts of 1 to solutions containing a fixed amount of ATP in the presence and absence of HPNPP

A solution of ATP (final concentration 15 μM) was prepared in each of 4 different cuvettes using an aqueous solution of HEPES buffer (5 mM, pH 7.0) as the solvent. To 3 cuvettes different amounts of HPNPP were added (final concentrations 20, 50, 100 μM). To the reference cuvette no HPNPP was added. Nile red (λ_{ex} = 570 nm, 2 μM) was added as fluorescence reporter. All four cuvettes were titrated with increasing amounts of **1**; after each addition the fluorescence intensity at 635 nm was measured. The excitation and emission slit width were kept constant at 5 and 10 nm, respectively, throughout the experiment. Figure S7 shows that in the presence of HPNPP the fluorescence intensity is higher at equal concentrations of **2** and that the difference gradually increases with increased HPNPP concentration. It reinforces our hypothesis that in the presence of HPNPP, more apolar domain is available in the system for the uptake of Nile Red compared to the system in which just ATP is present

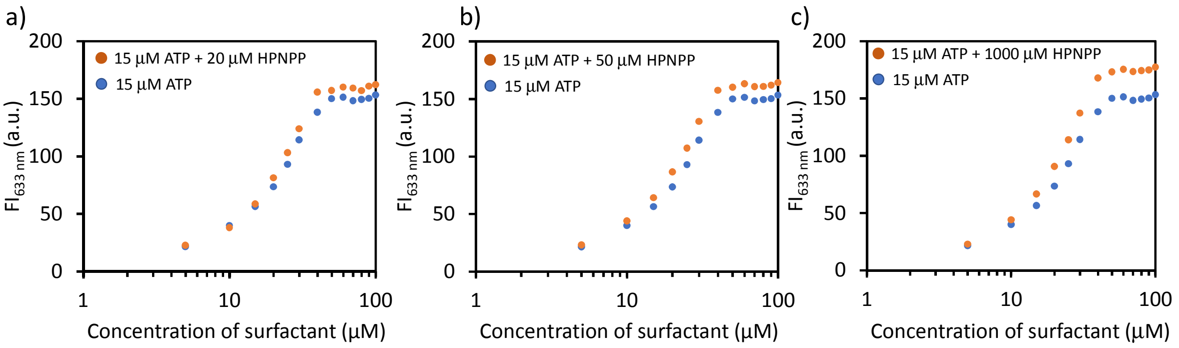

Figure S7. Emission intensity profiles for Nile Red (2 μM, λ_{ex} = 570 nm, λ_{em} = 635 nm) at increasing concentration of **1** and a constant concentration of ATP and in the presence and absence of different concentrations of HPNPP. [ATP] = 15 μM, [HPNPP] = 0, 20, 50, 100 μM, HEPES buffer (5 mM, pH 7), 25 °C.

In addition, we repeated the titration reported in Figure 3a at a concentration of HPNPP equal to 200 μM. A cac of around 40 μM was determined.

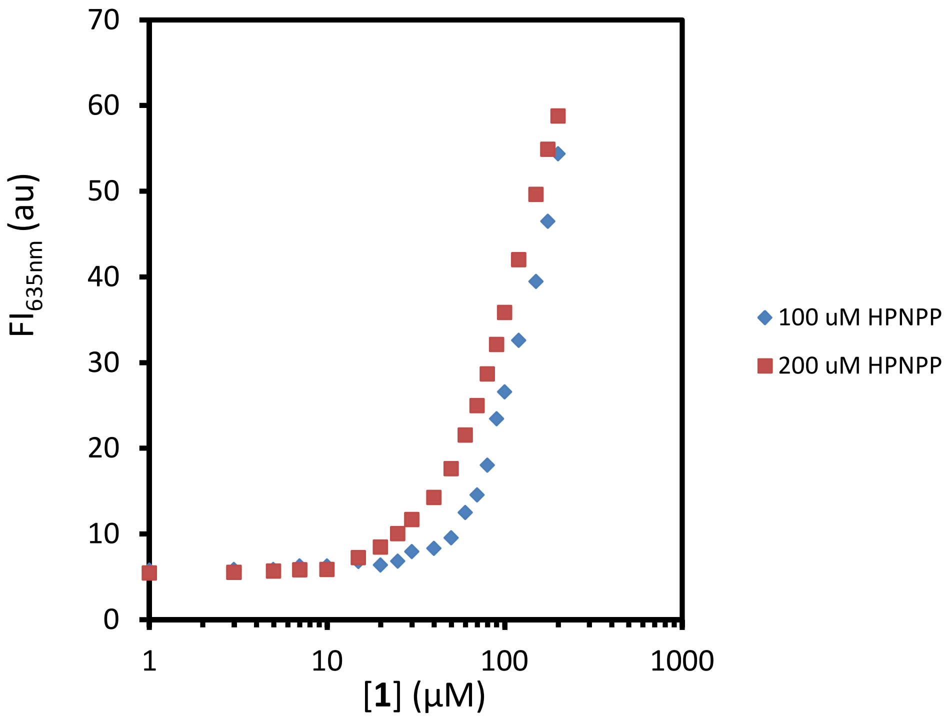

Figure S8. Fluorescence intensity of Nile Red (2 µM, λ_{ex} = 570 nm, λ_{em} = 635 nm) at increasing concentration of **1** and a constant concentration of HPNPP of 100 (blue) or 200 (red) µM. Experimental conditions: HEPES buffer (5 mM, pH 7), 25 °C.

8. DLS studies of the ATP-HPNPP system

Dynamic light scattering experiments were conducted to investigate the hydrodynamic diameter of the ATP-templated assemblies in the presence and absence of HPNPP. ATP (10 μM) was added to a solution of 100 μM **1** in aqueous buffer (5 mM HEPES buffer, pH 7.0) After 15 minutes to ensure stabilization of the system DLS spectra were recorded (blue lines). Then 200 μM HPNPP was added to the same sample and DLS data was recorded (orange line). Figure S9 reports four separate measurements.

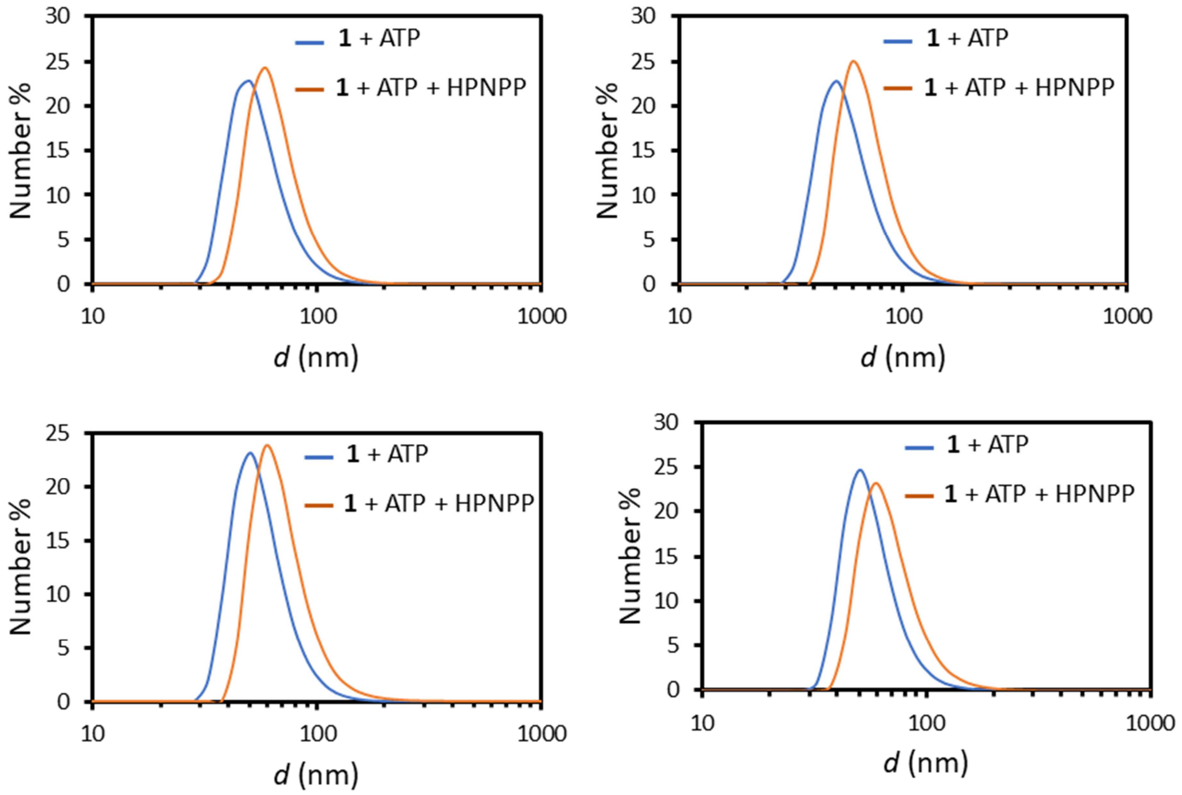

Figure S9. Hydrodynamic diameter of ATP-templated assemblies ([**1**] = 100 μM, [ATP] = 10 μM) in the absence (blue line) and presence (orange line) of HPNPP (200 μM) in aqueous buffer solution (HEPES, 5 mM; pH 7.0). The given data originate from 4 independent experiments to confirm the increment in size.

9. TEM analysis

TEM analysis was carried out by casting one drop of sample on the TEM grid and staining it with a 2% uranyl acetate solution. ATP-templated assemblies were prepared by adding 10 µM ATP to a solution of **1** (100 µM) in aqueous buffer (HEPES, 5 mM; pH 7.0) and leaving the sample 15 minutes. In other samples, HPNPP (200 µM) was added 15 minutes after the addition of 10 µM ATP was added to a solution of **1** (100 µM) in aqueous buffer solution (HEPES, 5 mM; pH 7.0). Sub-10 nm structures were excluded from our analysis.

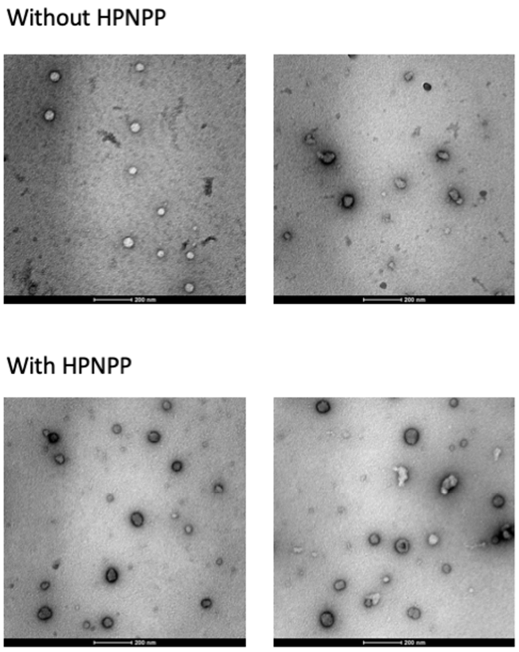

Figure S10. Additional representative TEM images of ATP-templated assemblies ([**1**]=100 µM, ATP 10 µM) in the presence and absence of HPNPP (200 µM) in aqueous buffer solution (HEPES, 5 mM; pH 7.0).

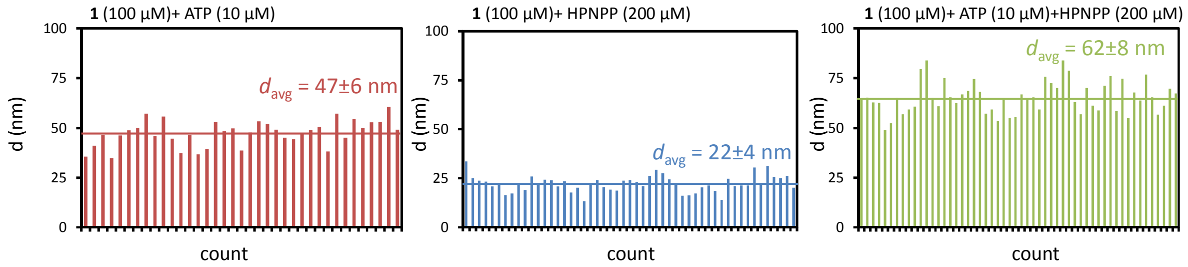

Figure S11. Analysis of structures visible in the TEM images that were used to construct the histogram depicted in Figure 3g.

10. In-situ acceleration of HPNPP catalysis by adding 1

The experiment depicted in Figure 4b was carried out as follows. Two cuvettes were prepared each containing a solution of **1** (100 μM), ATP (25 μM), and HPNPP (200 μM) in aqueous buffer (HEPES, 5 mM; pH 7.0). The kinetics of the reactions were followed by measuring the absorbance at 405 nm in time. After 15 min, to one cuvette an additional amount of **1** (25 μM) was added, whereas the other cuvette was left unaltered. Measurement of the absorbance at 405 nm was then continued for both cuvettes.

11. DLS study to explore the templating ability of AMP

Dynamic light scattering was used to investigate the hydrodynamic diameter of the templated assemblies in the presence of AMP. AMP (50 µM) was added to an aqueous buffered solution of **1** (100 µM, 5 mM HEPES buffer, pH 7.0) followed by DLS-measurement (blue line). The grey line is the control with only **1** (100 µM). An insignificant change in the DLS spectra indicates that AMP is unable to template assembly formation under the experimental conditions.

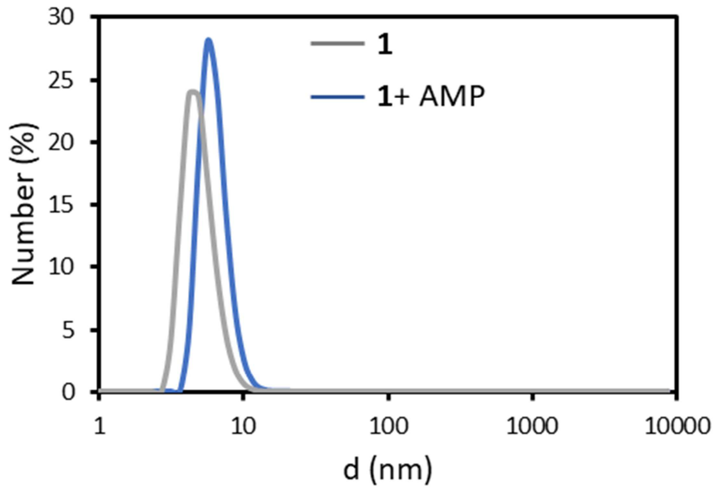

Figure S12. Hydrodynamic diameter of AMP templated vesicle (C16TACN·Zn^{2+} 100 µM, AMP 50 µM) in aqueous buffer solution (HEPES, 5 mM; pH 7.0).

12. DLS study of the ATP-AMP system

Dynamic light scattering experiments were used to investigate the hydrodynamic diameter of the ATP-templated assemblies in the presence and absence of AMP. ATP (10 μM) was added to a solution of **1** (100 μM) in aqueous buffer (5 mM HEPES buffer, pH 7.0) and left to equilibrate for 15 minutes before the DLS measurement was carried out (blue line). Then AMP (30 μM) was added to the same sample and the DLS measurement repeated (orange line). Three repetitions of the same experiment show that upon the addition of AMP the hydrodynamic diameter of the ATP- templated assemblies increases.

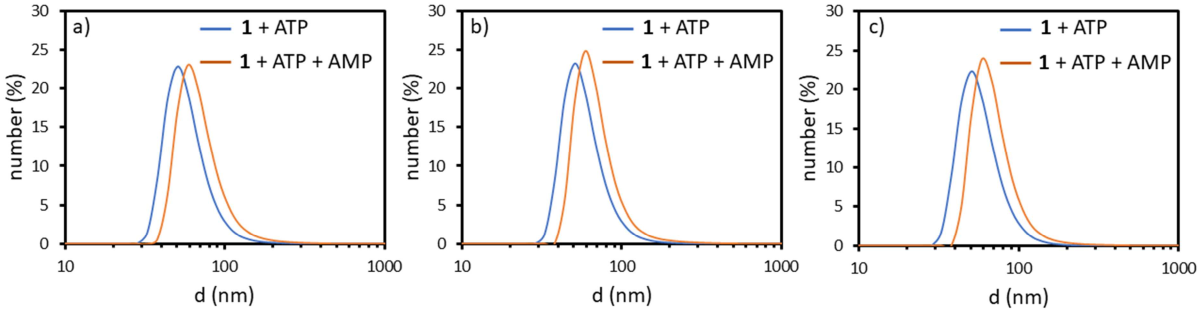

Figure S13. Hydrodynamic diameter of ATP-templated assemblies ([**1**]=100 μM, ATP=10 μM) in the absence (blue line) and presence (orange line) of AMP (30 μM) in aqueous buffer solution (HEPES, 5 mM; pH 7.0). Three separate experiments (a, b, c) were carried out.

13. FRET experiments

UV/vis absorption and photoluminescence spectra of Nile red (2µM) and NBD-GDDD[2] (3 µM) were measured in ethanol and buffer medium, respectively. Nile red by itself shows a poor solubility in buffer medium and thus we could not obtain a good signal. Nonetheless, we observed a significant overlap between the absorption spectrum of the Nile red with the emission spectrum of NBD-GDDD (Fig. S14). This observation suggests energy transfer from NBD (donor) to Nile red (acceptor) would be possible if they are in close proximity.

We studied acceptor emissions in templated and non-templated assemblies (Fig. 5 in the manuscript). A fixed amount of Nile red (2 µM) was added to a solution of **1** (100 µM) and ATP (10 µM) and excited at the absorption maximum of NBD (λ_{ex} =450 nm). A weak signal was observed (Fig. 5, blue trace), because Nile red has a poor absorption at this wavelength.

We studied the emission of the NBD donor under similar conditions. A strong fluorescence signal was observed with a maximum at 530 nm from a solution containing **1** (100 µM) and NBD-GDDD **1** (3 µM) (λ_{ex}=450 nm, Fig. 5, green trace). Just a small amount of NBD-GDDD was added to ensure that the majority of surfactant **1** remained in the unassembled state. The addition of Nile red (2 µM) to the same sample (Fig. 5, orange trace) leads to a decrease in the 530 nm emission (NBD) and an increase in the NR emission (633 nm). This indicates that FRET takes place between NBD-GDDD templated assemblies and NR embedded in the hydrophobic domain of these assemblies. In a second set of experiments, we added NBD-GDDD (3 µM) to preformed ATP templated assemblies (100 µM **1** + 10 µM ATP) in the presence of Nile red (2 µM) and excited at 450 nm (Fig. 5, red trace). The significant increase in fluorescence intensity at 633 nm (NR) and concomitant decrease at 530 nm (NBD) strongly suggests the colocalization of ATP and NBD-GDDD in the same assemblies. The stronger templating ability of ATP leads to an increased number of assemblies with a mixed ATP/NBD-GDDD surface composition. All experiments were carried out in HEPES (5 mM) buffered at pH 7 and T = 25 °C.

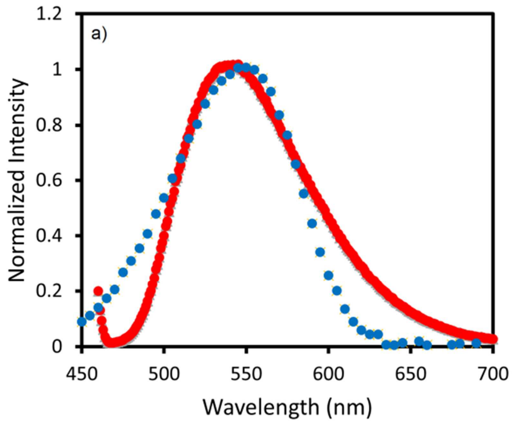

Figure S14. Normalized absorption (Nile red, blue dotted) and emission spectra (NBD-GDDD, red solid). Experimental conditions: λ_{ex} = 450 nm, slit= 10/10, [HEPES] = 5 mM, pH = 7, T=25°C.

14. Supplemental References

1. Solís Muñana, P. et al. *Angew. Chem. Int. Ed*. **2018**, *57*, 16469–16474.

2. Pieters, G.; Cazzolaro, A.; Bonomi, R.; Prins, L. J., *Chem. Commun*. , **2012**, *48*, 1916-1918.

3. Pieters, G.; Pezzato, C.; Prins, L. J., *J. Am. Chem. Soc*. **2012**, *134*, 15289-15292.

4. Pezzato, C.; Prins, L.J. *Nat. Commun.***2015**, 6: 7790.

5. Czescik, J.; Zamolo, S.; Darbre, T.; Mancin, F.; Scrimin, P. *Molecules*, **2019**, *24*, 2814.

6. Bonomi, R.; Cazzolaro, A.; Sansone, A.; Scrimin, P.; Prins, L.J. *Angew. Chem. Int. Ed.*, **2011**, *50*, 2307–12.
